# Diet-induced obesity in rats leads to a decrease in sperm motility

**DOI:** 10.1186/1477-7827-9-32

**Published:** 2011-03-11

**Authors:** Carla DB Fernandez, Fernanda F Bellentani, Glaura SA Fernandes, Juliana E Perobelli, Ana Paula A Favareto, André F Nascimento, Antonio C Cicogna, Wilma DG Kempinas

**Affiliations:** 1 Graduate Program in Cell and Structural Biology, Institute of Biology, University of Campinas (UNICAMP), Campinas, SP, Brazil; 2 Department of Morphology, Institute of Biosciences, UNESP - Univ Estadual Paulista, Botucatu, SP, Brazil; 3 Medical Clinic Department, Botucatu Medical School, UNESP - Univ Estadual Paulista, Botucatu, SP, Brazil

## Abstract

**Background:**

Obesity is rapidly becoming a worldwide epidemic that affects children and adults. Some studies have shown a relationship between obesity and infertility, but until now it remains controversial. Thus, the aim of the present study was to investigate the effect of high-fat diet-induced obesity on male reproductive parameters.

**Methods:**

In a first experiment, male Wistar rats were fed a high-fat diet (HFD) or standard chow (SD) for 15, 30 or 45 weeks, after which they were evaluated by adiposity index, serum leptin levels, reproductive organ weights and sperm counts. In a second experiment, rats received HFD or SD only for 15 weeks, long enough to cause obesity. Sexual hormones and sexual behavior were evaluated in these animals, as well as fertility after natural mating. Another group of rats was submitted to motility analysis and fertility evaluation after in utero insemination.

**Results:**

After 15, 30 or 45 weeks, HFD-fed animals presented significant increases in obesity index and serum leptin levels. Reproductive organ weights and sperm counts in the testis and epididymis were similar between the two groups at all timepoints studied. Sexual behavior was not altered by the diet regimen, and HFD fertility after natural mating was also similar to SD-fed animals. Intergroup testosterone levels were also comparable, but estradiol levels were increased in HFD rats. Furthermore, sperm quality was reduced in HFD animals as evidenced by their decreased percentage of sperm with progressive movement. This altered motility parameter was followed by a trend toward reduction in fertility potential after artificial in utero insemination.

**Conclusions:**

The results reported herein showed that obesity can affect sperm quality, by reducing sperm motility, without affecting other sperm parameters. The low sperm quality caused a slight reduction in fertility potential, showing that obesity may lead to impairment in male fertility.

## Background

Overweight and obesity constitute a health problem of increasing prevalence and present a major public health concern [1,2] that affects men and women, young and old [3]. These two statuses are often defined simply as a condition of abnormal or excessive fat accumulation in adipose tissue [4] arising from an imbalance between calories ingested versus calories expended [5]. The change in the average weight of the population is occurring quickly, and within a few generations the bell-curve of human-weight distribution has shifted toward greater weight [3].

Obesity is a risk factor for non-insulin-dependent diabetes, cardiovascular disease, osteoarthritis, some types of cancer, and certain reproductive and metabolic disorders [6]. It is also associated with disturbance in the hormonal milieu that can affect the reproductive system, which is clear in women who present reproductive disorders when obese [7,8]. However, in men this relationship is poorly characterized, due to the lower number of studies in the literature [2,9]. In recent years, some studies have associated the body mass index (BMI) with reproductive parameters in men, showing that increased BMI is related to poor semen quality [10], decreased sperm concentration [11], decreased normal-motile sperm cells and increased DNA fragmentation index [12]. On the other hand, some works showed little or no relation between obesity and sperm concentration [2,13], motility or morphology [2] in men, even when serum reproductive hormone levels are altered [2,13].

A small number of energy-balance genes are known to be essential for normal body regulation and a loss-of-function mutation in a single gene can lead to obesity in laboratory animals [14]. However, it does not explain obesity in the majority of the human population where no such genetic changes have been identified. If obesity were entirely genetic in causation, it would be difficult to explain the increased in prevalence of obesity over the last few decades. Contemporary diets are a major factor in the current obesogenic environment, and most human obesity could probably be assessed as being diet-induced [14]. Although genetic obesity models are useful for finding the role of endogenous neuropeptides in body weight control, the best parallels to human obesity are provided by the physiological model of diet-induced obesity (DIO) [14,15].

In diet-induced obese male mice decreases in sperm motility [16,17], fertilization rate [17] number of plugs and pregnancy rate [16], as well as increases in sperm DNA damage and sperm intracellular reactive oxygen species (ROS) have been reported [17]. However, Tortoriello and colleagues [18] found no impairment in the fertility of male DBA/2J mice after they were fed a high-fat diet. In studies of rats made obese by cafeteria feeding, a diminished number of ejaculations was observed [19]. Thus, in the literature, few studies report the effects of obesity on male fertility and sperm quality and the results are altogether less clear. Therefore the aim of this study was to determine the effect of high-fat diet-induced obesity on reproductive parameters in male rats.

## Methods

### Animals

Male (aged 5-6 weeks) and female (aged 11-12 weeks) Wistar rats were supplied by São Paulo State University Animal Center - UNESP - Botucatu/SP. During the experiment, animals were allocated individually into polypropylene cages, with laboratory grade pine shavings as bedding. Rats were maintained under controlled temperature (± 23°C) and lighting conditions (12L, 12D photoperiod, lights switched off at 07:00am). Rat chow and filtered tap water were provided ad libitum. Experimental procedures were in accordance with the Ethical Principles in Animal Research adopted by the Brazilian College of Animal Experimentation and were approved by the Biosciences Institute/UNESP Ethics Committee for Animal Research (protocol number 06/07).

### Experimental design

Male rats were randomly assigned to one of two different groups: the first was fed a high-fat diet, with a content of 20% fat (RC Focus 2413 Agroceres^®^, Rio Claro, São Paulo, Brazil) and the second received a standard diet with 4% fat content (RC Focus 1765 Agroceres^®^, Rio Claro, São Paulo, Brazil). The dietary regimen was adapted from previous studies [20,21].

The study was divided into two steps. In the first (experiment 1), rats were given the high-fat diet (HFD) or standard diet (SD) for 15, 30 or 45 weeks. In the second step (experiment 2) the animals were exposed to HFD or SD for a period of 15 weeks, long enough to increase adiposity index, which characterizes obesity.

### Experiment 1

#### Collection of tissue and organs

In this part of the study rats were given a high-fat diet (HFD) or standard diet (SD) for 15, 30 or 45 weeks. Rats were weighed every week, and food consumption was monitored daily. After each period of diet exposure, rats (n = 9-13/group/food exposure time) were slightly anesthetized with sodium pentobarbital ip (40/mg/kg), weighed and killed by decapitation. Blood was collected from the ruptured cervical vessels for determination of leptin levels. Adipose tissue was isolated and weighed from the epididymal, visceral and retroperitoneal pad. The right testis, epididymis, vas deferens, ventral prostate and seminal vesicle (without the coagulating gland) were removed and their weights (absolute and relative to body weights) were determined. Testis and epididymis were used for sperm counts.

#### Adiposity index

Adiposity index was determined by the sum of epididymal, visceral and retroperitoneal fat weights divided by body weight × 100, and expressed as adiposity percentage [22].

#### Daily sperm production per testis, sperm number and transit time in the epididymis

Homogenization-resistant testicular spermatids (stage 19 of spermiogenesis) in the testis were counted as described previously by Robb et al. [23], with adaptations adopted by Fernandes et al. [24]. Briefly, the testis, decapsulated and weighed soon after collection, was homogenized in 5 mL of NaCl 0.9% containing Triton × 100 0.5%, followed by sonication for 30 seconds. After a 10-fold dilution, one sample was transferred to Neubauer chambers (4 fields per animal), and late spermatids were counted. To calculate the daily sperm production (DSP), the number of homogenization-resistant spermatids was divided by 6.1, the number of days these spermatids are present in the seminiferous epithelium. In the same manner, caput/corpus and cauda epididymidis portions were cut into small fragments with scissors and homogenized, and sperm counted as described for the testis. The sperm transit time through the epididymis was determined by dividing the number of sperm in each portion by the DSP.

### Experiment 2

In this part of the study, animals were exposed to the high-fat or standard diet for 15 weeks, a period sufficient to characterize obesity. Rats were weighed every week, and food consumption was monitored daily. After the period of diet exposure, a group of rats (12-13/group) was assessed for sexual behavior and fertility outcomes after natural mating. Then, 15 days after the end of the sexual behavior test, rats were slightly anesthetized with sodium pentobarbital ip (40 mg/Kg), weighed and killed by decapitation. Blood was collected from the ruptured cervical vessels for determination of sexual hormone levels (testosterone, follicle stimulating hormone - FSH, luteinizing hormone - LH, estradiol). Three fat deposits - epididymal, visceral and retroperitoneal - were removed and weighed, as already described. Semen was collected from the right and left deferens ducts to evaluate sperm motility and sperm morphology, respectively. The right testes were collected for *in vitro *testosterone assay. Another group of rats (11/group), exposed to the different diets as previously described, had fertility tested by *in utero *artificial insemination.

#### Serum testosterone, FSH, LH and estradiol levels

The serum was obtained by centrifugation (2400 rpm, for 20 minutes at 4°C) and the concentrations of testosterone, estradiol, luteinizing hormone (LH) and follicle-stimulating hormone (FSH) were determined by the technique of double antibody radioimmunoassay. Testosterone doses were accomplished by using the TESTOSTERONE MAIA^® ^kit (Biochem Immuno System). The LH and FSH doses used specific kits supplied by the National Institute of Arthritis, Diabetes and Kidney Diseases (NIADDK, USA). All samples were dosed in the same assay to avoid inter-assay errors. The intra-assay error was 3.4% for LH, 2.8% for FSH and 4% for testosterone.

#### Evaluation of sexual behavior and natural mating

At the end of the 15-week diet exposure, male rats from each experimental group were placed individually in polycarbonate crystal boxes, measuring 44 × 31 × 16 cm, 5 min before introduction of one adult female rat in natural estrus (sexually receptive) determined by vaginal smear. The animals were observed in the dark period of the cycle in a separate room under dim red light, and all sexual behavior tests were performed 2-4 h after the beginning of the dark period. For the next 40 min the following parameters were evaluated: latency to the first mount, intromission and ejaculation; number of intromissions until the first ejaculation; latency of the first post ejaculatory intromission; number of post ejaculatory intromissions; and number of ejaculations [25,26]. The males that did not mount in the initial 10 min were considered sexually inactive.

After the sexual behavior test the couples were kept together for an additional 4 hours. The animals that had been deemed inactive were tested one more time, for fertility, with different females in estrus. At the end of the afternoon males and females were separated and vaginal smears were collected. The day on which sperm were found in the smear was determined to be gestational day 0 (GD0); females were killed 20 days later to evaluate fertility.

#### Sperm motility and morphology

Immediately after euthanasia, the right vas deferens was collected. Sperm were obtained with the aid of a syringe and needle, through internal rinsing with 1.0 mL of modified HTF medium (Human Tubal Fluid, IrvineScientific) at 34°C. A Makler counting chamber (Sefi-Medical, Haifa, Israel) warmed to 34°C was loaded with a small aliquot of sperm solution. Sperm motility evaluation was performed by the same person throughout the study and was assessed by visual estimation (100 spermatozoa per animal, in duplicate) under a phase-contrast microscope (Leica DMLS) at 200X magnification. Spermatozoa were classified as: immotile, motile without progression and motile with progressive movement. Sperm were also removed from the left vas deferens by internal rinsing with 1.0 mL of saline formol, with the aid of a syringe and needle. To analyze sperm morphology, smears were prepared on histological slides that were left to dry for 90 min and observed in a phase-contrast microscope (400 × magnification) [27], and 200 spermatozoa were analyzed per animal. Morphological abnormalities were classified into two general categories pertaining to head morphology (without curvature, without characteristic curvature, pin head or isolated form, i.e., no tail attached) and tail morphology (broken, isolated, i.e., no head attached or rolled into a spiral) [28].

#### Intratesticular testosterone concentration

The right testis of each animal was removed and decapsulated, and the parenchyma was sliced into ~50-mg pieces. Each piece was weighed and placed into a 1.5-ml micro tube containing 1.0 ml of Medium 199 (M199). The M199 was buffered with 0.71 g/L sodium bicarbonate (NaHCO3) and 2.1 g/L Hepes, and contained 0.1% BSA (bovine serum albumine) and 25 mg/L soybean trypsin inhibitor, pH 7.4. Testosterone concentration was assessed by incubating parenchyma in duplicate, for 2 h at 34°C [29]. After centrifugation (5 min, 10.000 × *g*), medium was frozen at -70°C until testosterone assay, which was performed as described previously.

#### *In utero *artificial insemination

Because rats produce and ejaculate an excess of qualitatively normal sperm, artificial *in utero *insemination of a fixed critical number of sperm has been suggested as a means of increasing the sensitivity of a toxicant-induced decrease in sperm quality in the rat [30]. According to this technique, a fixed number of sperm collected from the cauda epididymis is inseminated directly into the uterus allowing evaluation of sperm quality, without the interference of other factors such as alterations of the sexual behavior pattern and number of sperm available for ejaculation [31].

A cohort of females (n = 40) was synchronized with a single subcutaneous injection of 80 μg of luteinizing releasing hormone (LHRH) agonist (Sigma Chemical Co., St Louis, Missouri), 115 hours prior to the insemination. Shortly after the room lights were turned off on the day of proestrus, the synchronized females were paired with sexually experienced, vasectomized males of proven sterility for 1 h. Receptive females (that exhibited lordosis) were selected for insemination. The isolation and preparation of proximal cauda sperm for insemination were the same as described previously [32,33], with the following adaptations. Briefly, the sperm were released from the proximal cauda by nicking the tubule with a n 11 scalpel and allowed to disperse in 2 ml of modified HTF medium (Human Tubular Fluid, IrvineScientific^®^). After 5 min of dispersion, a sperm aliquot was diluted 1:10 with fixative (10% formalin in PBS) and counted using a Neubauer chamber. Within 15 min, each uterine horn was injected with a volume containing 5 × 10^6 ^sperm [32]. One female was inseminated per male. All inseminations were performed while the recipient female was in a surgical plane using a mix of ketamine and xylazine anesthesia. The bifurcation of the uterine horns was exposed through a low, midventral incision. Fine curved forceps were used to elevate each horn while the insemination volume was injected through the wall of each horn via an 18-gauge i.v. catheter attached to a 1.0-ml syringe. Each injection site was cauterized immediately upon withdrawal of the needle. When insemination was completed, the abdominal musculature was sutured. Females were killed 20 days later to evaluate fertility.

#### Fertility evaluation

On the GD20 the females that had been naturally and artificial inseminated were killed by decapitation. After collection of the uterus and ovaries the numbers of corpora lutea, implants, reabsorptions and live and dead fetuses were determined. From these results the following parameters were calculated: gestation rate: number of pregnant females/number of inseminated females × 100; fertility potential (efficiency of implantation): implantation sites/corpora lutea × 100; rate of preimplantation loss: [number of corpora lutea - number of implantations/number of corpora lutea] × 100; and rate of postimplantation loss: [number of implantations - number of live fetuses]/number of implantations × 100.

### Statistical Analysis

Two-way ANOVA for independent groups followed by the *post hoc *Tukey test were performed for comparison of results among the experimental groups in experiment 1. For experiment 2 Student t test or nonparametric Mann-Whitney test were used according to the characteristics of each variable. Differences were considered significant when p < 0.05.

## Results

### Experiment 1

Throughout the course of the study, the mean food intake of HFD rats was significantly lower than the mean food intake of SD rats, in all experimental periods. In contrast, total caloric consumption between the groups was similar at 15 weeks (HFD = SD) and elevated at 30 and 45 weeks (HFD > SD) (Table 1). Body weight, fat deposits, adiposity index and serum leptin increased significantly in a time-dependent manner in obese and control animals but were higher in the obese group (HFD > SD) (Table 2).

**Table 1:** Daily mean food and calories consumption in rats from SD and HFD groups

	*15 weeks*		*30 weeks*		*45 weeks*	
	SD	HFD	SD	HFD	SD	HFD
Daily food intake (g)	28.6 ± 0.4^Aa^	22.5 ± 0.3^ABb^	27.1 ± 0.3^Ba^	23.1 ± 0.3^Ab^	26.5 ± 0.2^Ba^	22.0 ± 0.2^Bb^
Daily calories intake (kcal)	90.5 ± 1.2^Aa^	91.8 ± 1.2^ABa^	85.5 ± 1.0^Ba^	94.4 ± 1.0^Ab^	83.8 ± 0.7^Ba^	89.7 ± 0.7^Bb^
*N*	11	13	12	11	11	9

Data expressed as means ± SEM. The capital letters refer to comparison between times, while lower case letters refer to the comparison between SD and HFD groups. Different letters indicate a significant difference (p < 0.05, Two-Way ANOVA, post hoc Tukey Test)

**Table 2:** Body weight, fat weighs and leptin levels in rats from SD and HFD groups

	*15 weeks*		*30 weeks*		*45 weeks*	
	SD	HFD	SD	HFD	SD	HFD
Final body weight (g)	468.36 ± 5.58^Aa^	502.31 ± 8.41^Ab^	533.08 ± 8.44^Ba^	583.09 ± 13.00^Bb^	567.82 ± 13.18^Ba^	644.22 ± 20.43^Cb^
Fat deposits (g)						
Epididymal	7.85 ± 0.31^Aa^	10.67 ± 0.69^Ab^	8.90 ± 0.73^Aa^	12.87 ± 1.29^Ab^	12.77 ± 1.05^Ba^	16.84 ± 1.08^Bb^
Visceral	4.98 ± 0.36^Aa^	7.08 ± 0.59^Ab^	5.62 ± 0.43^Aa^	9.46 ± 0.95^Ab^	9.32 ± 0.76^Ba^	13.17 ± 1.21^Bb^
retroperitoneal	7.06 ± 0.30^Aa^	11.56 ± 1.38^Ab^	8.79 ± 1.03^Aa^	14.71 ± 1.82^Ab^	13.83 ± 0.90^Ba^	22.65 ± 2.34^Bb^
Adiposity index (%)*	4.22^Aa^	5.78^Ab^	4.35^Aa^	6.26^Ab^	6.30^Ba^	8.09^Bb^
Leptin levels (ng/mL)	2.88 ± 0.28^Aa^	5.42 ± 0.34^Ab^	4.98 ± 0.41^Ba^	8.17 ± 0.80^Bb^	7.32 ± 0.77^Ca^	10.92 ± 0.93^Cb^
*N*	11	13	12	11	11	9

Data expressed as means ± SEM. The capital letters refer to comparison between times, while lower case letters refer to the comparison between SD and HFD groups. Different letters indicate a significant difference (p < 0.05, Two-Way ANOVA, post hoc Tukey Test)

*Adiposity index values were transformed in arc sen prior to statistical analysis and they are expressed as mean

Reproductive organs weights did not show any differences between HFD and SD groups in all treatment periods, excepting the relative weight of empty seminal vesicle which was lower in HFD animals than in SD after 45 weeks of diet exposure (Table 3). When animals from different timepoint were compared, differences were found even among control groups (Table 3).

**Table 3:** Absolute and relative organs weight in rats from SD and HFD groups

	*15 weeks*		*30 weeks*		*45 weeks*	
	SD	HFD	SD	HFD	SD	HFD
*Absolute organs weight*						
Testis (g)	1.88 ± 0.04^Aa^	1.82 ± 0.06^Aa^	1.74 ± 0.11^Aa^	1.91 ± 0.06^Aa^	1.94 ± 0.05^Aa^	2.12 ± 0.11^Aa^
Epididymis (mg)	666.73 ± 14.77 ^Aa^	671.69 ± 14.87 ^Aa^	629.33 ± 34.61 ^Aa^	668.55 ± 18.35 ^Aa^	646.45 ± 14.75 ^Aa^	683.78 ± 40.45 ^Aa^
Vas deferens (mg)	99.73 ± 2.81^Aa^	98.41 ± 6.29^Aa^	116.17 ± 4.97^Aa^	114.00 ± 5.52^Aa^	119.73 ± 4.14^Ba^	130.11 ± 3.71^Ba^
Ventral prostate (mg)	478.91 ± 43.43^ABa^	542.54 ± 37.14^ABa^	588.75 ± 29.11^Aa^	556.55 ± 32.37^Aa^	418.18 ± 39.68^Ba^	417.89 ± 32.18^Ba^
Seminal vesicle full (g)	1.24 ± 0.08^Aa^	1.37 ± 0.09^Aa^	1.36 ± 0.09^Aa^	1.48 ± 0.07^Aa^	1.46 ± 0.07^Aa^	1.59 ± 0.07^Aa^
Seminal vesicle empty (mg)	578.36 ± 38.34^Aa^	658.62 ± 30.83^Aa^	728.83 ± 59.15^Ba^	745.00 ± 34.09^Aa^	703.73 ± 33.48^ABa^	680.89 ± 43.02^Aa^
*Relative organs weight*						
Testis (g/100 g)	0.40 ± 0.01^Aa^	0.36 ± 0.01^Aa^	0.34 ± 0.02^Ba^	0.33 ± 0.01^Aa^	0.34 ± 0.01^Ba^	0.32 ± 0.02^Aa^
Epididymis (mg/100 g)	141.44 ± 3.41^Aa^	133.93 ± 3.03^Aa^	121.46 ± 5.48^Ba^	115.52 ± 4.93^Ba^	114.21 ± 2.90^Ba^	107.49 ± 8.21^Ba^
Vas deferens (mg/100 g)	21.16 ± 0.62^Aa^	19.68 ± 1.25^Aa^	21.97 ± 0.98^Aa^	19.59 ± 0.86^Aa^	21.23 ± 0.96^Aa^	20.37 ± 0.97^Aa^
Ventral prostate (mg/100 g)	101.90 ± 9.58^Aa^	107.74 ± 7.01^Aa^	111.11 ± 5.43^Aa^	96.49 ± 6.76^Aa^	74.30 ± 7.54^Ba^	65.72 ± 6.09^Ba^
Seminal vesicle full (g/100 g)	0.26 ± 0.02^Aa^	0.27 ± 0.02^Aa^	0.26 ± 0.02^Aa^	0.26 ± 0.01^Aa^	0.26 ± 0.01^Aa^	0.25 ± 0.01^Aa^
Seminal vesicle empty (mg/100 g)	122.55 ± 7.92^Aa^	130.96 ± 5.28^Aa^	140.21 ± 11.65^Aa^	127.93 ± 5.87^Aa^	123.90 ± 5.06^Aa^	105.85 ± 6.34^Aa^
*N*	11	13	12	11	11	9

Data expressed as means ± SEM. The capital letters refer to comparison between times, while lower case letters refer to the comparison between SD and HFD groups. Different letters indicate a significant difference (p < 0.05, Two-Way ANOVA, post hoc Tukey Test)

There was no statistically significant difference between SD and HFD groups related to the number of mature spermatids in the testis and daily sperm production (Table 4). When animals were compared in relation to time, the number of mature spermatids in the testis was lower in 30 and 45-weeks animals in both experimental groups. In the epididymis the number of spermatozoa in caput/corpus was similar between SD and HFD groups in all periods of diet exposure, but in epididymal cauda of animals fed HFD for 30 weeks there was an increase in sperm number compared to SD animals in this timepoint (Table 4). The sperm transit time did not show any difference between SD and HFD either in epididymal caput/corpus or cauda (Table 4).

**Table 4:** Sperm counts in rats from SD and HFD groups

	*15 weeks*		*30 weeks*		*45 weeks*	
	SD	HFD	SD	HFD	SD	HFD
Sperm number in the testis (×10^6^)	291.70 ± 6.02^Aa^	294.02 ± 8.25^Aa^	228.42 ± 12.30^Ba^	248.05 ± 13.56^Ba^	233.92 ± 9.64^Ba^	239.54 ± 19.73^Ba^
Daily sperm production (×10^6^/testis/day)	47.82 ± 0.99^Aa^	48.20 ± 1.35^Aa^	37.45 ± 2.02^Ba^	40.66 ± 2.22^Ba^	38.35 ± 1.58^Ba^	39.27 ± 3.24^Ba^
Sperm number in the caput/corpus epididymis (×10^6^)	171.32 ± 7.97^Aa^	171.77 ± 8.2^Aa^	142.44 ± 14.01^Aa^	152.26 ± 6.13^Aa^	172.58 ± 8.99^Aa^	162.86 ± 18.06^Aa^
Sperm transit time in the caput/corpus (days)	3.62 ± 0.17^Aa^	3.58 ± 0.15^Aa^	4.22 ± 0.26^ABa^	3.83 ± 0.22^Aa^	4.52 ± 0.21^Ba^	4.26 ± 0.42^Aa^
Sperm number in the cauda epididymis (×10^6^)	347.61 ± 17.72^Aa^	330.30 ± 13.3^Aa^	243.82 ± 31.46^Ba^	320.94 ± 16.77^Ab^	291.78 ± 14.44^ABa^	281.57 ± 24.61^Aa^
Sperm transit time in the cauda (days)	7.35 ± 0.41^Aa^	6.85 ± 0.2^Aa^	6.6 ± 0.81^Aa^	8.02 ± 0.43^Aa^	7.74 ± 0.50^Aa^	7.30 ± 0.68^Aa^
*N*	11	13	12	11	11	9

Data expressed as means ± SEM. The capital letters refer to comparison between times, while lower case letters refer to the comparison between SD and HFD groups. Different letters indicate a significant difference (p < 0.05, Two-Way ANOVA, post hoc Tukey Test)

### Experiment 2

As occurred in experiment 1, HFD rats showed a statically significant increase in adiposity index, body weight and weights of fat deposits (data not shown) after 15 weeks of diet exposure.

Sexual behavior, assessed in this experiment, was not exhibited by some of the animals (2/13 in SD and 2/14 in HFD). Among those who presented sexual behavior, HFD animals showed an increase (p < 0.05) in latency to the first mount and a decrease (p < 0.05) in the number of intromissions after the first ejaculation. The other parameters evaluated in the test were similar between the two groups (Table 5).

**Table 5:** Sexual behavior of rats fed SD or HFD during 15 weeks

	SD	HFD
Latency to the first mount (s)	56.45 ± 12.06 (n = 11)	133.3 ± 35.93* (n = 12)
Latency to the first intromission (s)	110.45 ± 24.09 (n = 11)	169.67 ± 44.98 (n = 12)
Number of intromissions until the first ejaculation	19.94 ± 2.01 (n = 11)	14.50 ± 1.36 (n = 12)
Latency to the first ejaculation (s)	664.00 ± 68.82 (n = 8)	946.67 ± 123.91 (n = 12)
Latency to the first post-ejaculatory intromissions (s)	952.00 ± 66.01 (n = 8)	1234.64 ± 142.30 (n = 11)
Number of post-ejaculatory intromissions	25.75 ± 2.2.07 (n = 8)	14.82 ± 2.75* (n = 11)
Number of ejaculations	2.88 ± 0.23 (n = 8)	2.17 ± 0.30 (n = 12)
N	11	12

Data expressed as means ± SEM. Student t test. *p < 0.05

Serum testosterone, FSH and LH levels were similar between SD and HFD animals; furthermore, parenchyma testosterone was not affected in HFD rats compared to SD animals. On the other hand, estradiol levels were significantly higher in HFD-fed animals (Table 6).

**Table 6:** Serum sexual hormone levels and parenchyma testosterone of rats fed SD or HFD during 15 weeks

	SD	HFD
Serum testosterone (ng/mL)	1.93 ± 0.31	2.89 ± 0.55
Serum LH (ng/mL)	0.99 ± 0.27	0.91 ± 0.27
Serum FSH (ng/mL)	10.20 ± 0.71	11.97 ± 1.74
Estradiol (pg/mL)	8.69 ± 0.38	11.11 ± 0.91*
Parenchyma (ng/mg)	69.50 ± 7.72	70.56 ± 8.19
N	13	14

Data expressed as means ± SEM. Mann-Whitney test. *p < 0.05

**Figure 1:**
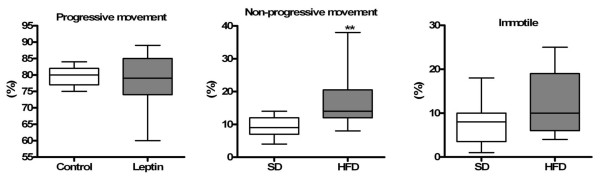
**Sperm motility**. Sperm motility of rats from SD and HFD groups. Values expressed by median. Mann-Whitney test. **p < 0.001.

Results obtained from the morphological assessment of spermatozoa indicated that the percentages of both abnormal and normal sperm were similar between SD and HFD rats [SD = 94 (91-97)% and HFD = 93 (91-95)%, median (Q1-Q3) values for normal sperm]. The percentage of sperm with progressive movement was significantly diminished (p < 0.05) and the percentage of sperm without progressive movement was elevated (p < 0.05) in the HFD group when compared to SD animals (Figure 1).

The gestational rate was one hundred percent in both groups after natural mating, and thus there were no differences between SD and HFD groups regarding any fertility parameters (Table 7). After the *in utero *artificial insemination, the gestational rate was 100% in SD group and 78% in HFD. Fertility potential showed a tendency to decrease whereas the pre-implantation loss rate tended to increase in HFD animals, but without statistical significance (Table 7).

**Table 7:** Fertility parameters after natural mating and *in utero *artificial insemination of rats fed SD or HFD during 15 weeks

	Natural Mating		*In utero *insemination	
	SD	HFD	SD	HFD
Fertility potential (%)^a^	100 (92.15 - 100)	100 (85.7 - 100)	91.89 (77.43 - 100)	75.00 (65.39 - 95.46)
Body weight of dams (g)^b^	356.67 ± 10.67	355.88 ± 6.51	314.80 ± 12.58	286.16 ± 12.43
Number of corpora lutea ^b^	12.25 ± 0.45	13.15 ± 0.37	12.90 ± 0.74	11.86 ± 0.40
Number of implantation sites ^b^	11.67 ± 0.69	12.38 ± 0.40	10.9 ± 0.55	8.71 ± 1.41
Number of fetuses per litter ^b^	11.25 ± 0.66	10.85 ± 1.00	10.7 ± 0.58	7.86 ± 1.41
Preimplantation loss (%)^a^	0 (0 - 7.85)	0 (0 - 14.3)	8.12 ( 0 - 22.32)	25.00 ( 4.55 - 34.62)
Postimplantation loss (%)^a^	0 (0 - 7.1)	0 (0-7.1)	0 ( 0 - 0)	0 ( 0 - 0)
*N*	12	13	10	7

^a^Data expressed as median (Q1 - Q3). ^b^Data expressed as mean ± S.E.M. Mann-Whitney test. p > 0.05

## Discussion

It is believed that with the increasing prevalence of sedentary lifestyles and dietary changes, obesity is emerging, in turn, as an important cause of adverse health outcomes, including male infertility [34]. Data from different population studies show an inverse relationship between BMI (body mass index) and fertility [10,11], although the mechanism by which fertility is affected is still unclear [35].

In an attempt to achieve deeper knowledge about obesity, several animal models have been developed, among which rodent models of diet-induced obesity (DIO) may provide the best parallels in relation to human obesity [14,15,18]. In this study, an obesity model induced by high-fat diet consumption was chosen. It is cited in the literature as causing obesity in a variety of mammals including nonhuman primates, dogs, pigs, hamsters, squirrels, mice [36], and rats [36,37].

The high-fat diet used in the present study was effective in promoting obesity, as demonstrated by an increased adiposity index in association with a higher body weight. This condition was exacerbated by long-term ingestion of a high-fat diet. The obese state was related to higher caloric intake in the HFD group at 30 and 45 weeks; however, obesity also was evident at 15 weeks even in the absence of higher caloric consumption by HFD rats. This condition may have been due to a high feeding effectiveness ratio in the hyperlipidic diet. There is evidence that high fat consumption is not accompanied by a proportional increase in its oxidation. This phenomenon favors the deposition of lipids such as triacylglycerol in adipose tissue, leading to an increase in body weight [38,39]. Unfortunately, there is no established threshold to differentiate obesity from overweight in animal models, such as those established by the WHO for humans [40]. It should be taken into account that in this study, the adiposity index was at least 25% higher in obese animals than in the control group at all experimental moments.

The adiposity index was calculated as the sum of the weights of the fat pads divided by body weight; this quotient represented an estimation of body fat. Results found herein for control animals are in accordance with other studies in the literature [22,41] that used similar methods for fat measurement. In animal models of genetic obesity, the adiposity index is higher [41] than that found in DIO animals, because the first case represents very severe obesity.

Leptin, a hormone produced mainly by adipocytes, is involved in controlling body weight by increasing both satiety and energy expenditure [18,35,42]. Leptin is also related to the reproductive system [3,43] through both stimulatory and inhibitory effects [44,45]. The leptin concentration is related to the amount and distribution of body fat [6] such that the heavier the body weight the higher the leptin concentration in human and rodents [42]. The elevated serum leptin levels observed in the current study are likely a consequence of the increased fat accumulation. This result corroborates other studies in the literature that show high leptin levels in models of rodent DIO obesity [16,18,35,44,46,47].

Few studies in the literature relate organ weight to obesity. In Zucker rats, a genetic model of obesity, obese animals show no difference in the weights of testes or ventral prostate compared with lean rats [48]. Similarly, DIO male mice exhibited no changes in the average weight of the testis or epididymis [16]. These data are in accordance with the results of present study.

When animals were compared in relation to time, differences were found among different timepoints, even in control animals which occurred probably due to aging process. Aging in male rats is known to be associated with some testicular alterations that result in decreased spermatogenesis and steroidogenesis [49] and gradual reduction in sperm production [50].

The number of spermatids present in the testis and the total DSP are important indicators of male fertility potential [51]. In this study, the consumption of HFD for 15, 30 or 45 weeks did not affect any of these parameters and the sperm concentration in the epididymal caput-corpus. The epididymal cauda sperm concentration was not altered in animals fed HFD for 15 and 45 weeks, which corroborates studies of male HFD-fed mice [16,17]. On the other hand, Sprague-Dawley rats fed a high-fat diet from 21 to 90 days old presented a reduced sperm concentration [35]. Some works show a reduction in sperm concentration in obese and overweight men [9,11] while another found no alterations in sperm concentration related to BMI [2]. Animals fed HFD for 30 weeks showed an increase in epididymal cauda sperm concentration, which was probably due to a non-significant increase in sperm transit time in this segment. However, this change did not alter fertility. Although sperm transit time through the epididymis plays an important role in the maturation of spermatozoa sperm quality and fertility potential are not harmed when transit time through the epididymis is delayed [33,52].

In general, sexual behavior among animals fed the high-fat diet was normal, despite the delay to start the test. The normal sexual behavior observed in these animals is in concordance with their normal serum testosterone levels, which were similar to those of rats fed a standard diet. A reduction in testosterone levels expected on account of the higher leptin level in HFD animals from first experiment was not observed. Contrary to the results in the present study, Sprague-Dawley rats fed HFD from weaning to 90 days had a reduction in testosterone levels [35], and male mice fed HFD (for 9 weeks) showed a trend toward reduction in testosterone levels compared to the control group [17]. Among men, overweight and obesity is frequently related to diminished testosterone levels [2,11,13], a decrease proportional to the degree of obesity [53]. The adiposity gain seen in the animals was not sufficient to produce a significant diminution in the testosterone levels, perhaps because the obesity installed was not severe; however, it was enough to provoke a significant increase in serum estradiol levels, in accordance with results found by Vigueras-Villaseñor and colleagues [35]. Obesity is associated with increased estradiol levels also in men [2,11].

Sperm motility is one of the most important parameters used in the evaluation of sperm quality [54-56]. This sperm parameter is acquired during sperm transit through the epididymal duct [57-61]. Epididymal histophysiology and acquisition of sperm motility are dependent on the presence of androgen [62,63]. In the present study the percentage of sperm with progressive motility was reduced despite normal levels of testosterone. Male mice rendered obese by consuming a high-fat diet also showed a diminished percentage of motile sperm without presenting alterations in testosterone levels [16]. In men, an inverse relationship between BMI and the number of normal-motile sperm was observed in some clinical studies [9,12], but was not found in others [2,13].

Alterations in motility parameters may lead to an inefficient sperm penetration of cervix mucus [64-66], impairing the ability of sperm to reach the oocyte. In addition, a high percentage of sperm with progressive motility is related to a high fertilization index [67]. Despite this, even with the decrease in progressive motility, the fertility after natural mating was not altered in HFD animals. Corroborating this result, DIO male DBA/2J mice (24% of fat in diet) did not show any alteration in fertility in relation to lean animals [18]. Contrarily, another study found a significant reduction in fertility after natural mating of diet-induced (60% of fat in diet) obese male C57BL/6J mice [16]. Another important sperm parameter for evaluating male fertility is sperm morphology [68] because it may indicate cytotoxic events [69]. The absence of morphological alterations in obese animals indicates a high probability that obesity did not negatively affect spermatogenesis. In the literature there is an interesting observation that spermatogenesis is affected only in males with extreme obesity [70].

Given the impairment in sperm motility and the lack of effects on fertility after natural mating in the present study, we chose to utilize artificial insemination, a technique that excludes the influence of excess sperm ejaculated [71], to detect some impairment of fertile capacity of sperm with impaired motility. Fertility potential after artificial insemination showed a trend toward reduction. It is important to take into consideration that although the fertility potential was not significantly affected, there was a reduction of around 20% in this parameter indicating a possible reduction in fertile capacity of sperm. Similar results were found in mice by Bakos and colleagues [17], who observed a reduction in percentage of fertilized oocytes, using sperm from diet-induced obese mice (22% fat in the diet). The absence of statistical significance in the fertility results may be due to high reproductive competence of rats, which need to show a large impairment of sperm quality to be considered infertile [72].

In summary, the results reported herein show that the HFD treatment causes obesity in rats. The obese animals present a low sperm quality, elucidated by the decreased percentage of sperm with progressive movement that tends to impair fertility without affecting other sperm parameters. The reproductive capacity of male rats is known to be higher than that of men; therefore, the decrease in sperm quality seen in obese rats was not sufficient to significantly alter their fertility, whereas such a decrease in quality may be enough to alter fertility among human males. Since obesity is a growing health problem worldwide, additional studies are needed to investigate more deeply the relationship between obesity and male infertility.

## Competing interests

The authors declare that they have no competing interests.

## Authors' contributions

All authors participated in the design, interpretation of the studies, analysis of the data and review of the manuscript; CDBF, GSAF, FFB, APF and JEP conducted the experiments; CDBF and WDGK wrote the manuscript. This study represents part of CDBF Ph.D. thesis presented to the State University of Campinas, under the advisory of WDGK. All authors read and approved the final manuscript.
